# The clinical efficacy of single-hole punch excision combined with intralesional steroid injection for nodular keloid treatment: a self-controlled trial

**DOI:** 10.1038/s41598-024-60670-x

**Published:** 2024-04-29

**Authors:** Bingbing Liu, Haoying Lin, Minghai Zhang

**Affiliations:** Department of Dermatology, Chaohu Hospital Affiliated to Anhui Medical University, Chaohu, 238000 Anhui China

**Keywords:** Single-hole, Punch excision, Nodular keloid, Steroid, Intralesional injection, Symmetry, Recurrence, Diseases, Medical research

## Abstract

There are many methods to treat keloid, including various excision operations, laser, injection and radiotherapy. However, few studies have explored the effectiveness of single-hole punch excision in keloid treatment. This study aimed to investigate the efficacy and safety of lateral punch excision combined with intralesional steroid injection for keloid treatment through self-control trial. In this self-controlled trial, 50 patients meet the diagnosis of nodular keloid, and try to choose left–right symmetrical control, one skin lesion in the control group (50 skin lesionsin total) and the other in the observation group (50 skin lesions in total).The keloids in the treatment group were initially treated with punch excision combined with intralesional steroid injection, followed by injection treatment alone. Keloids in the control group received intralesional steroid injection alone. The Vancouver Scar Scale (VSS) of the keloid before and after the punch excision was evaluated; the keloid scores at different time points and the number of injection treatments required in both groups were compared, and adverse reactions were observed. The effective rate of the observation group was 86.0%, which was significantly higher than that of the control group (66.0%), and the recurrence rate of 22% was lower than that of the control group (χ^2^ = 4.141,63417), all of which were statistically significant (all *P* < 0.05). At the end of treatment, the VSS and total injection times in the observation group were significantly lower than those in the control group (t = 5.900,3.361), with statistical significance (*P* < 0.01). The combination of single-hole punch excision and intralesional steroid injection is an effective method to treat multiple nodular keloids, shortening the treatment course of tralesional steroid injection without obvious adverse reactions.

## Introduction

Keloid is caused by skin injury (such as infection and trauma), and it is a common fibro proliferative disease in clinic^1,2^. It is common in teenagers and young women, and it can cause itching and discomfort^3–5^, and even affect the appearance, which has attracted much attention^1^. According to the characteristics of keloids, they are classified as: (1) fresh nodular keloids; (2) superficial disseminated keloid; (3) mature (non-growing) keloids; (4) multiple keloids^6^. Surgical resection is the main method to treat single giant keloid, but traditional surgery for multiple nodular keloids is generally not used because of its high risk of trauma^7,8^ . Therefore, minimally invasive and non-invasive treatment is the main research directions for multiple nodular keloids^9–11^. At present, non-invasive treatment methods include radiotherapy and intralesional steroid injection, among which intralesional steroid injection is relatively cheap and the curative effect is not necessarily worse than radiotherapy^12–15^.However, the above therapeutic effects are still limited and cannot meet the higher requirements of patients. Recently, there has been a report on the treatment of multiple keloids by minimally invasive porous resection combined with local injection, and the effect is remarkable^15,16^. Due to the fact that this method requires many holes to be drilled on the surface of the scar, resulting in significant trauma and slow recovery in the later stages, it may cause adverse effects on the patient. By improving the method, since 2022, our department has also achieved good results in the treatment of multiple nodular keloids by single-hole excision of scar tissue with fewer traumas combined with intralesional steroid injection for keloid treatment. The report is as follows.

## Clinical data and methods

### Study design and ethics

In this study, a total of 50 patients with symmetrical keloids who were admitted to our institution between August 2022 to December 2023 and try to choose one skin lesion in the control group (50 skin lesionsin total) and the symmetrical other in the observation group (50 skin lesions in total). Approval was obtained from the ethics committee of Anhui Medical University (KYXM-2208-013). We certify that the study was performed in accordance with the 1964 declaration of HELSINKI and later amendments. Written informed consent was obtained from all the participants prior to the enrollment of this study.

### Clinical data

**Figure 1:**
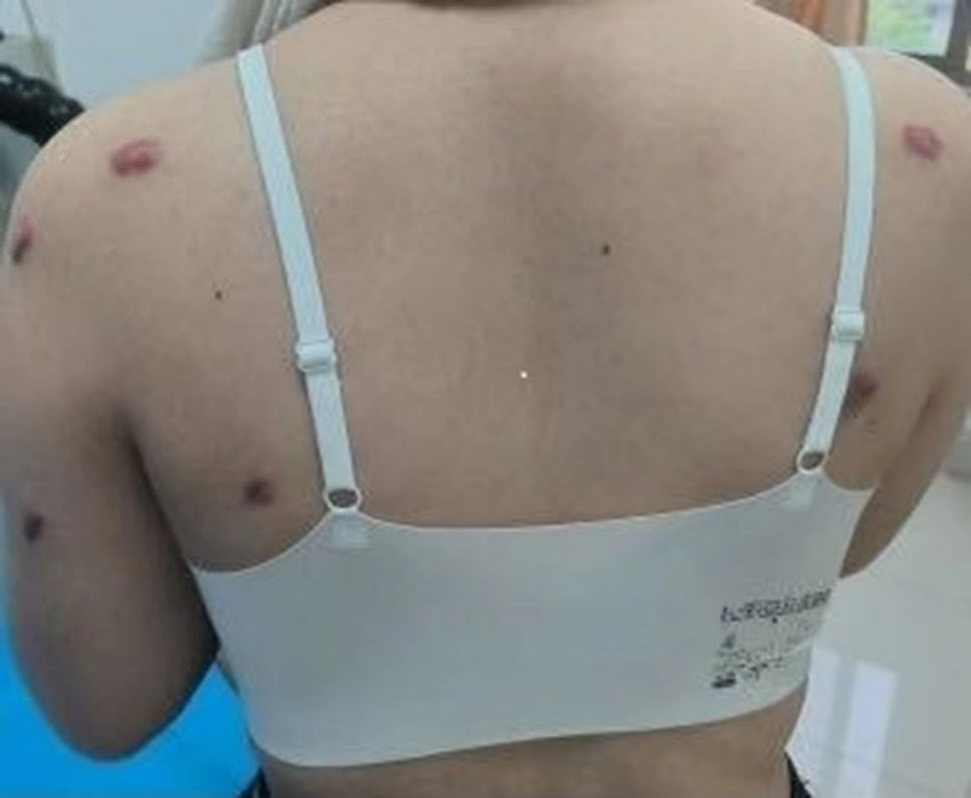
The typical presentation of symmetric multiple nodular scarring.

From August 2022 to December 2023, 50 patients with symmetrical multiple nodular keloids with 100 sites were collected from the Department of Dermatology, Chaohu Hospital, Anhui Medical University. The typical presentation of symmetric multiple nodular scarring is shown in Fig. 1.

### Inclusion and exclusion criteria

The inclusion criteria were as follows: all patients (1) met the diagnostic criteria of multiple nodular keloids; (2) with the keloids are symmetrical; (3) who did not receive any other treatments within 3 months. The exclusion criteria of using Intralesional steroid injection and therapy and punch excision were as follows: all patients (1) with fungal or bacterialinfection at the keloids or system; (2) with cutaneous atrophy, ulcers or malignant change in keloids; (3) with serious immune system diseases and organ damage. (4) with hypersensitivity to betamethasone or other glucocorticoid drugs or any of the ingredients in this product.

### Methods

**Figure 2:**
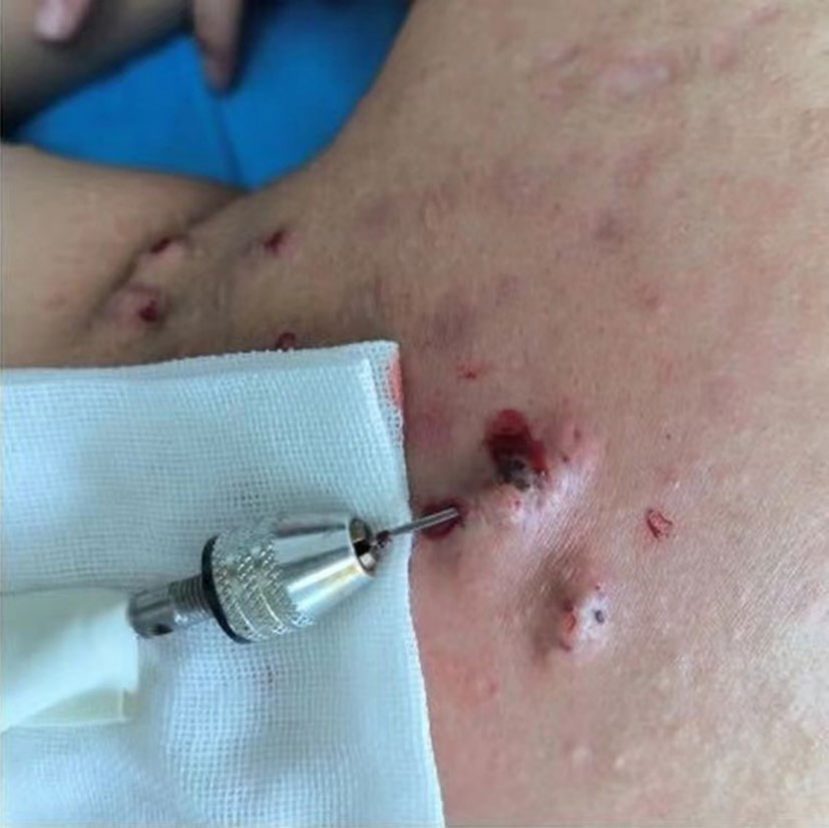
The method of punch excision: the needle was inserted from the side of the keloid by electric circular rotation (the inner diameter of the needle hole was 1.0 mm, and the outer diameter was 1.1 mm), and all the scar tissues were transferred from different directions in one hole as far as possible until only the superficial skin tissue formed a dermal vascular network flap.

Before operation, all patients underwent routine blood tests, hemagglutination tests and screening for infectious diseases (hepatitis B, hepatitis C, syphilis and AIDS). Self-left and right controls were adopted. Adopting random drawing lots if the left one was included in the control group (50skin lesionsin total), then the right one was included in the observation group (50skinlesions in total). Control group: only the mixture of 2% lidocaine injection (specification 5 ml) and compound betamethasone^17,18^ (produced by Shanghai Schering-Plough Pharmaceutical Co., Ltd., national medicine standard word J20080062, specification: 1 ml) injection was injected into the keloids once a month until the scar was flat. Observation group: After injecting the mixture of 2% lidocaine injection and compound betamethasone into the keloids, the needle was inserted from the side of the keloid by electric circular rotation (the inner diameter of the needle hole was 1.0 mm, and the outer diameter was 1.1 mm)(Fig. 2), and all the scar tissues were transferred from different directions in one hole as far as possible until only the superficial skin tissue formed a dermal vascular network flap, and the pressure was bandaged^19^ for 3 days, which was the same as that of the control group.

### Evaluation indexes

Follow-up through outpatient for one year to evaluate the curative effect, and the evaluation criteria of clinical efficacy are as follows: (1)Cure, the pain disappears, the keloid is completely softened and flattened, and it is soft and has no indurations, and there is no recurrence after one year of follow-up after treatment; (2)Remarkable results, the symptoms such as itching disappeared or significantly alleviated, and 60–70% of the keloid area softened and flattened, and there was no recurrence after one year of follow-up; (3)Effective, symptoms such as itching and color have been alleviated, and keloid have improved; (4)Ineffective, after treatment, the symptoms such as keloid have not changed or itching have been alleviated and have reached the standard of cure and remarkable effect. Recurrence: Recurrence one year after the end of treatment. The effective rate = [(number of cured cases + number of remarkable cases)/total number of cases] × 100%. The Vancouver Scar Scale (VSS)^20,21^ was used to evaluate the scar before and after treatment. Including color, blood vessels, softness, thickness, and pain and itching, with a total score of 0–18. The higher the score, the more serious the scar is. Adverse reactions: mainly including purpura, punctate necrosis, hypopigmentation, pigmentation, epidermal atrophic telangiectasia, etc.

### Statistical methods

Statistical analyses were performed using the Statistical Package for the Social Sciences software. Categorical variables are expressed as the number of cases and percentages (n [%]), which were assessed using the chi-squared test. The Shapiro–Wilk test was used to test whether the samples of the numerical variables conformed to a normal distribution: those that conformed to a normal distribution are expressed as (x ± s) and assessed using the t-test. *P* value of < 0.05 was considered statistically significant.

### Ethical approval

Approval was obtained from the ethics committee of Anhui Medical University (KYXM-2208-013). We certify that the study was performed in accordance with the 1964 declaration of HELSINKI and later amendments. Written informed consent was obtained from all the participants prior to the enrollment of this study.

## Results

### Curative effects

**Figure 3:**
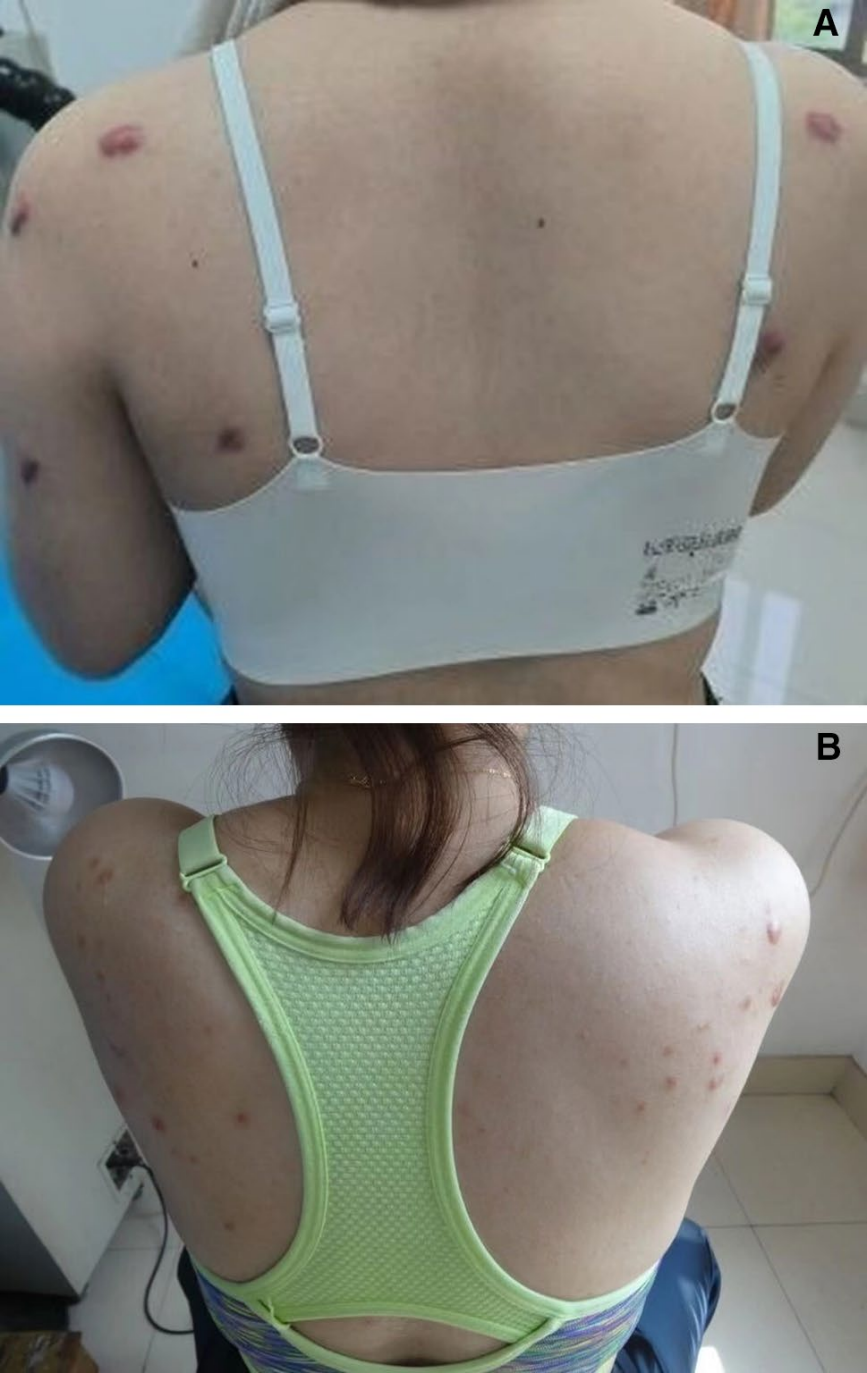
(**A**) Before treatment: the left side is drilling combined with hormone therapy, and the right side is hormone therapy (**B**) After 1 year of treatment: the left side is drilling combined with hormone therapy, and the right side is hormone therapy.

Comparison of treatment effects between the two groups. The left trephine side is obviously better than the right hormone treatment side, as shown in Fig. 3A, B.After one year follow-up, the effective rate in the observation group was 86.0%, which was significantly higher than that in the control group (66.0%), and the recurrence rate was 22%, which was lower than that in the control group (χ2 = 4.141, 3.417), with statistical significance (all *P* < 0.05) (Table 1).

**Table 1:** Comparison of curative effect between two groups.

Groups	Control	Observation	χ2	*P*
Cure	23	31		
Remarkable	10	12		
Effective	17	7		
Ineffective	0	0		
Recurrence	23	11		
Effective rate (%)	66.00	86.00	4.141	< 0.05
Recurrence rate (%)	46.00	22.00	3.417	< 0.05

### The vancouver scar score and total injection times

After 1 year, the VSS and total injection times in the observation group were significantly lower than those in the control group (t = 5.900, 3.361), with statistical significance (*P* < 0.01) (Table 2).

**Table 2:** The Vancouver scar score and total injection times between the 2 Groups.

Groups	Control	Observation	t	*P*
VSS of Before treatment	12.84 ± 2.46	12.70 ± 2.31	0.293	> 0.05
VSS of after treatment	3.60 ± 1.36	2.14 ± 1.11	5.900	< 0.01
injection times	1.32 ± 0.53	0.99 ± 0.45	3.361	< 0.01

### Adverse reactions

In the observation group, there were 2 pigment changes and 1 capillary dilatation. In the control group, there were 3 pigment changes and 2 capillary dilatation. The incidence of adverse reactions in the observation group was 6% lower than that in the control group (10%), and there was no statistical significance (χ2 = 1.190, *P* > 0.05) (Table 3).

**Table 3:** Comparison of adverse reactions between the 2 groups.

Groups	Control	Observation	χ2	*P*
Purpura	0	0		
Necrotize	0	0		
Infection	0	0		
Pigment change	3	2		
Capillary dilatation	2	1		
Skin atrophy	2	1		
Total	7	4		
Adverse reactions (%)	14	8	1.190	> 0.05

## Discussion

Keloid is a benign proliferative tumor of skin fibrous tissue^22^. Its main characteristics are that the lesions are higher than the normal skin surface, persistently proliferating, tough in texture, with or without pain or itching, which seriously affects the quality of life of patients^3,4,23^. Surgery is one of the effective methods for treating keloids, but it needs to be combined with other treatments, otherwise the recurrence rate is high, and studies have shown that the efficacy of injection therapy is not necessarily worse than radiotherapy, and it is cheap and suitable for hospitals with limited medical conditions. Therefore, surgery combined with local injection is also a common clinical treatment method^24–27^. For multiple nodular keloids, surgical treatment is not recommended, mainly including (1) direct excision, more damage, high risk; (2) if nucleotomy is used, the incision often has to be more than half of the circumference of the keloid or even more in order to remove the keloid nucleus, and the skin trauma is also large, and the same surgical risk exists; (3) many facets, small scars, small incisions, long surgery time, and great pain, which is not easy to be accepted by the patients. Therefore, carrying out minimally invasive surgical treatment is a new way to treat such keloids.

In this study, the minimally invasive lateral flap method combined with local injection therapy was used to treat nodular keloid scars, which has the advantages of low trauma, high safety and low recurrence rate. In this study, a rotary needle with an inner diameter of 1.0 mm and an outer diameter of 1.1 mm was used to enter the needle on one side of the scar, and all the scar tissue was excised from the same point of entry in different directions parallel to the skin surface, which preserved the integrity of the scarred skin to the maximum extent. The principle is the same as scar nucleotomy^28–31^. Although it is not as clean as surgical excision, it has the advantage of almost no trauma to the skin tissue above the scar. This study showed that the efficacy of minimally invasive flap hole removal of scar tissue was significantly better than that of local injection treatment alone, and the recurrence rate was also significantly lower than that of scar injection treatment alone, with statistically significant differences (all P < 0.05). After treatment, the Vancouver score was significantly lower than that of the local injection group (*P *< 0.05). Minimally invasive treatment is virtually non-invasive to the skin tissue above the scar, with no thermal damage and little damage to the skin's blood vessels, so there are few side effects and no skin necrosis. Even the incidence of adverse effects of local injection combined with lateral hole transfer was lower than that of the local injection group alone, but it was not statistically significant.

The strengths of this study are mainly the following two points: (1) the patients themselves were controlled before and after treatment to control interfering factors to avoid the lack of persuasive results due to individual differences; (2) this study destroys as much as possible the dense and hard fibrous tissue network inside the keloid, while maximizing the preservation of the integrity of the skin above the keloid. In contrast to the multiple-hole punch, the single-hole punch has less skin trauma, faster recovery, and is more acceptable. However, this study has some limitations, first, in the collected cases, the selected cases were samples from the area where the hospital was located, the selected sample range was narrow, the sample size was small, and there was subjectivity in the collected data; second, during the operation process, it was not possible to accurately control the amount of injected medication as well as the length of the time interval between injecting the medication and single-hole perforation, and it was not possible to control the depth of the perforation with the same intensity; third, this study used only the Vancouver Scar Scale for efficacy assessment both before and after treatment, using only a single subjective scar scale, not considering the scar from the patient's perspective, and is greatly influenced by the subjective factors of the assessor, lacking a certain degree of objectivity. Fourth, this study examined a novel therapy for nodular keloids, but it remains unknown whether this therapy is specifically equally safe and effective for keloids of greater extent. Finally, despite the less invasive and safer nature of single-hole perforation compared with minimally invasive multiple-hole perforation, whether the effectiveness of the former for nodular keloids is superior to the latter remains unknown and requires further clinical trials.

Although single-hole punch excision combined with intradermal steroid injection therapy has been found to be safe and effective, further studies are needed to examine the cost-effectiveness, acceptability, and compliance of single-hole punch excision and to explore its potential impact on the pharmacokinetics of topical corticosteroid administration. Further validation of the superimposed benefits of single-hole punching for intracorticosteroid injection in the treatment of keloids requires further design of more rigorous clinical trials.

## Conclusion

In a word, at present, the treatment of multiple nodular keloids is mainly local injection, but it takes a long time to make the scar tissue shrink. If the minimally invasive lateral hole-turning method combined with local injection is used, it has the advantages of less trauma, high safety and low recurrence rate, which is worthy of clinical application.
